# A 14-Year Cohort of Candidemia in the Pediatric Population in a Tertiary Center in Jerusalem: Clinical Characteristics, Antifungal Susceptibility, and Risk Factors for Mortality

**DOI:** 10.3390/jof9121171

**Published:** 2023-12-06

**Authors:** Maya Korem, Asher Taragin, Danna Dror, Violeta Temper, Dina Averbuch

**Affiliations:** 1 Faculty of Medicine, Hebrew University of Jerusalem, Jerusalem 91120, Israeldanna.reg@gmail.com (D.D.);; 2 Department of Microbiology and Infectious Diseases, Hadassah Medical Center, Jerusalem 91120, Israel; 3 Pediatric Infectious Diseases, Pediatric Division, Hadassah Medical Center, Jerusalem 91120, Israel

**Keywords:** pediatric, candidemia, risk factors, fluconazole, mortality

## Abstract

*Candida* spp. can cause bloodstream infection and is associated with significant mortality. The proportion of fluconazole-resistant *Candida* non*-albicans* has increased over the years, and empirical fluconazole maybe inappropriate. In this retrospective study, we analyzed clinical characteristics, antifungal resistance patterns, and mortality in children with candidemia treated at a tertiary medical center in Jerusalem between 2009 and 2022. A total of 122 children developed 127 candidemia episodes with 132 *Candida* isolates. Half the episodes occurred in immunocompromised children. Septic shock was present in 27 (21.3%). *Candida* non-*albicans* was responsible for 71/132 (56.5%) episodes; 16/132 (12.1%) of isolates were fluconazole-resistant. The rate of *Candida* non-*albicans* was significantly higher in fluconazole-resistant episodes (90 vs. 50.5%, *p* = 0.02). Prolonged severe neutropenia and previous fluconazole exposure were more frequent in fluconazole-resistant episodes. Thirty-day mortality was 25 (19.7%). Greater mortality, as shown by multivariate analysis, was associated with candidemia contracted in the pediatric intensive care unit (PICU), previous use of azoles or carbapenems, and in the presence of shock. In conclusion, mortality rates in our study were higher than those previously reported. In suspected infection associated with factors which we found to increase the probability of mortality—PICU admission, shock, and earlier azole or carbapenems exposure—empirical antifungals should be considered.

## 1. Introduction

Invasive candidiasis (IC) or candidemia is among the most common causes of nosocomial bloodstream infections (BSI) [1]. Annual incidence of candidemia is 15.7 to 17.5 per 100,000 in infants under one year of age in the United States, compared with 0.8 per 100,000 in children aged 1 to 19 years [2]. Untreated, candidemia has a mortality of over 60% [3]. Even with treatment, mortality in children is high—between 10 and 26% [4,5], justifying the continuous research on this topic, including attempts to prevent IC with novel antifungal vaccines [6,7] and the use of combination therapy practiced in some centers [8,9].

Major risk factors for IC in children are immune deficiency (following, for example, hematopoietic cell transplantation (HCT), steroid therapy, or chemotherapy); hospitalization in a pediatric intensive care unit (PICU); and central venous catheterizations (CVC). Advances in supportive care, and ICU treatment modalities, together with the ability of *Candida* spp. to form a biofilm on invasive medical devices, has led to an increase in the population at risk for IC [10]. Less common risk factors in children are damage to the gastrointestinal tract mucosa (due, for example, to perforation or abdominal surgery), parenteral nutrition, previous use of broad-spectrum antibiotics, renal failure requiring dialysis, and mechanical ventilation [2].

*Candida albicans* is the most common cause of candidemia, although recent years have seen a significant increase in *Candida* non-*albicans* species [11,12]. The most commonly reported of these in children are *Candida glabrata* (currently *Nakaseomyces glabrata*) and *Candida parapsilosis*, followed by *Candida tropicalis* and *Candida krusei*. Their increasing incidence limits treatment options because of fluconazole resistance, mainly among *Nakaseomyces glabrata* and *C. krusei* isolates [4].

Echinocandins are a first-line treatment for candidemia in adults and children. Fluconazole, however, is considered an acceptable alternative as initial therapy in patients who are not critically ill and are considered unlikely to have fluconazole-resistant *Candida* species [1]. In clinical practice, fluconazole is frequently used to treat candidemia because of its favorable side effects profile, low cost, and good oral bioavailability [1].

The main risk factors for fluconazole resistance among *Candida* species in adults are previous fluconazole exposure, chronic renal disease, and neutropenia [13]. Data on risk factors for fluconazole resistance in children are limited. Because of the increase in candidemia due to *Candida* non-*albicans*, an empirical treatment with fluconazole may be inappropriate and can adversely affect the prognosis of patients with candidemia. In this study, we examined local epidemiology, antifungal resistance patterns, and risk factors for mortality in the pediatric population with candidemia in our hospital, with the aim of improving treatment decisions and patient outcomes.

## 2. Materials and Methods

### 2.1. Study Design

This study was an observational retrospective analysis of data retrieved from medical records of children (<18 years old) with a diagnosis of candidemia, treated at the Hadassah-Hebrew University Medical Center in Jerusalem between 2009 and 2022. This center is a tertiary hospital, divided into two campuses and providing medical care for 475,000 children in the Jerusalem area, as well as those referred from other parts of Israel or abroad. The study was approved by the Hadassah Medical Center Independent Review Board (0142-20-HMO).

### 2.2. Data Collection

Data on candidemia episodes at the time of the first positive blood culture were collected from electronic medical files and included demographics, background comorbidities, hospitalization course, presence of risk factors, laboratory and microbiology data, imaging studies, treatment, and outcome (Table 1). The inclusion criteria were as follows:

(1) blood culture yielding *Candida* spp.;
(2) patient aged 0–18 years;
(3) clinical picture consistent with infection, such as fever, deterioration in patient condition.

Patients with single positive blood culture yielding *Candida* spp., (without a compatible clinical picture, and subsequently negative blood cultures) who recovered without treatment were excluded.

### 2.3. Microbiology Data

Species identification and antifungal susceptibility testing was described elsewhere [11]. Briefly, during 2009–2012, *Candida* species were identified using CHROMagar *Candida* (HiLabs, Rehovot, Israel) and API ID 32 C (bioMerieux, Marcy Iletoile, France). *Candida tropicalis* and *Candida albicans* were identified solely based on CHROMagar Candida, whereas all other species underwent further testing using API ID 32 C. Since 2012, isolates have been identified mainly by matrix-assisted laser desorption ionization time-of-flight mass spectrometry (MALDI TOF-MS, VITEK MS, bioMerieux, Marcy Iletoile, France). Susceptibility testing was performed as part of routine patient care, using the E-test method according to manufacturer’s instructions (bioMerieux, Marcy Iletoile, France) until 2012, and since then has been performed with the VITEK2 yeast antimicrobial susceptibility testing (AST) cards (bioMerieux, Marcy Iletoile, France). Susceptibility results were interpreted according to the Clinical and Laboratory Standards Institute (CLSI) [14]. The MIC endpoints of the E-test were elevated to the next 2-fold dilution concentration, which matched the dilution schema of the microdilution method. *Candida krusei* was considered universally resistant to fluconazole [15]. In cases in which no clinical or efficacy data were available, epidemiological cut-off values (ECVs) were used, according to the CLSI M60 document [14]. Resistant isolates were defined as non-susceptible by CLSI breakpoints (MIC > breaking point of susceptibility), or non-wild-type (WT) when ECVs were used (MIC > ECV) [11]. In cases of *Nakaseomyces glabrata*, isolates with fluconazole MIC > 32 μg/mL were considered resistant, whereas susceptible-dose-dependent (SDD) isolates with MIC ≤ 32 μg/mL were considered susceptible, for analysis purposes.

### 2.4. Definitions

Candidemia episode: Isolation of single or multiple *Candida* spp. in blood. Isolation of two different *Candida* spp. from blood ≥48 h apart, was considered a separate episode.

Poly-candidemia: Isolation of two different *Candida* spp. in blood less than 48 h apart.

Fluconazole-resistant candidemia episode: Candidemia episode with at least one fluconazole-resistant *Candida* spp.

Nosocomial candidemia: Candidemia onset ≥ 48 h after hospitalization.

Previous hospitalization: Hospitalization lasting more than 48 h in past 3 months.

Severe neutropenia: Absolute neutrophil count (ANC) below 500 cells/µL.

Profound neutropenia: ANC below 100 cells/µL.

Prolonged severe neutropenia: Severe neutropenia lasting for more than seven days.

Chronic disseminated candidiasis: Candidiasis affecting liver, spleen, kidneys or progressive retinal exudates or vitreous opacities on ophthalmologic examination [16] or endocarditis.

Previous systemic antibacterial therapy: Antibiotics administered a month before candidemia.

Previous antifungal therapy: Antifungals administered a month before candidemia.

Inappropriate treatment: Antifungal treatment prescribed at candidemia onset to which the isolated pathogen (or one of the pathogens in cases of poly-candidemia) demonstrated in vitro resistance.

Mortality was recorded within 30 days from the first positive culture that yielded *Candida.*

### 2.5. Statistical Analysis

Fluconazole-resistant candidemia episodes were compared to fluconazole-susceptible episodes, and episodes that ended with mortality were compared to episodes that did not end with mortality. Analysis of continuous variables was performed using the parametric *t*-test and the non-parametric Mann–Whitney test according to the presence or absence of normal distribution, respectively. Pearson’s Chi-squared/Fisher’s exact tests were used for testing the association between two categorical variables. Variables found to be significant in univariate analysis were included in the multivariate analysis, which was performed using the logistic regression model with the stepwise forward, likelihood ratio approach. Statistical analysis was performed with SPSS software for Windows version 25 (IBM Corp., Armonk, NY, USA). A two-sided *p*-value of ≤0.05 was considered statically significant for all statistical tests.

## 3. Results

### 3.1. Clinical Characteristics (Table 1)

Clinical characteristics of the candidemia episodes examined are presented in Table 1. During the study period, there were 127 candidemia episodes in 122 children (117 children experienced 1 episode, and 5 had 2). Poly-candidemia was seen in four episodes, (3.1%) and the total number of *Candida* isolates was 132. Most infections were nosocomial, reflected by a prolonged median time from hospitalization to candidemia (12 days, IQR 5, 28). Immunosuppression was present in about half of all episodes, with the proportion higher during the later years of the study (21/51, 41.1% in 2009–2013; 45/76, 59.2% in 2014–2022), mainly following HCT and in hematological malignancies (43/127, 33.8%). A high proportion (41.7%) of episodes occurred in the PICU. Septic shock was present in 21.3%. Candidemia duration was short (median of 1 day, IQR 0, 5). In most episodes (89.8%), antibacterial therapy had been administered in the preceding month, predominantly beta-lactams.

No differences were found between fluconazole-susceptible and -resistant episodes in demographics, background, or clinical or laboratory parameters. The rates of exposure to fluconazole (3/10, 30% vs. 16/111, 14.4%) and prolonged severe neutropenia (3/10, 30% vs. 15/111, 13.6%) were numerically higher in patients with fluconazole-resistant vs. -susceptible episodes.

### 3.2. Microbiological Characteristics (Table 2)

**Figure 1:**
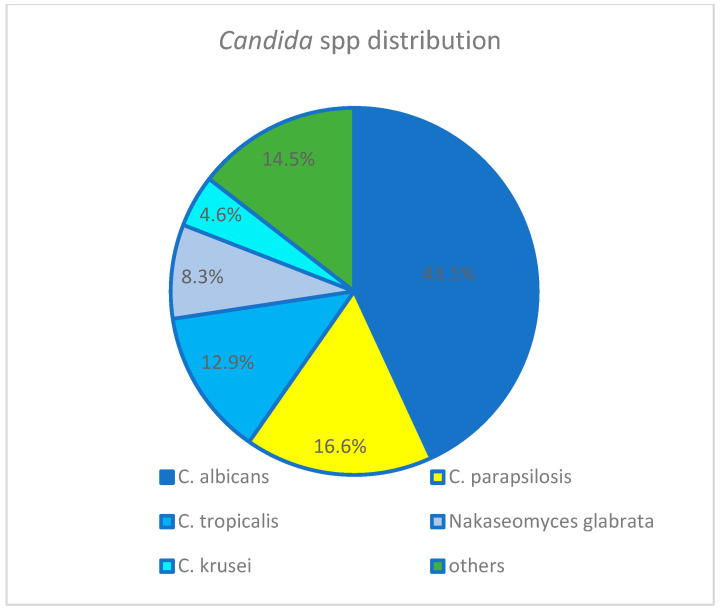
Others: *Candida lusitania* (4), *Candida lipolytica* (1), *Candida dubliniensis* (6) *Candida pelliculosa* (4), *Candida guillermondi* (3), *Candida kefyr* (1).

The predominant pathogen among 132 *Candida* isolates (Figure 1) was *C. albicans,* followed by *C. parapsilosis*. The fluconazole resistance rate among the 132 isolates was 12.1%: 14/93 (15%) during 2009–2017 and 4/39 (10.2%) during 2018–2022. The rate of *Candida* non-*albicans* was significantly higher in fluconazole-resistant episodes (90 vs. 50.5%, *p* = 0.02).

### 3.3. Treatment and Outcome (Table 1 and Table 3)

Inappropriate treatment was given in 9/127 (7.1%) of episodes. A CVC was present in 104 episodes (81.9%), and removed following candidemia in 46 of them (36.8%).

The 30-day mortality was 25/127 (19.7%), with an increase observed in the later period 2009–2013: 5/51, 9.8%; 2014–2022: 20/76, 26.3%). Factors significantly associated with 30-day mortality were abdominal surgery, total parenteral nutrition (TPN), candidemia in PICU, time from hospital admission to candidemia, previous antifungals (specifically, use of azoles), previous carbapenems or vancomycin use, presence of shock, and longer candidemia duration (Table 1). A multivariate logistic regression model showed candidemia in PICU, previous use of azoles and carbapenems, and presence of shock to be independent predictors of 30-day mortality (Table 3).

## 4. Discussion

This study describes the clinical characteristics of invasive candidiasis episodes in a pediatric population admitted to a large tertiary-care medical center in Jerusalem over 14 years.

We found *Candida* non-*albicans* spp. to predominate in pediatric invasive candidiasis (56.5 vs. 43.5% *C. albicans*, respectively), similar to trends observed in adult patients [12], although species distribution differs between pediatric and adult populations. *C. parapsilosis* predominates in children, and *Nakaseomyces glabrata* in adults. This difference may be attributed to distinct host characteristics and lesser use of azole prophylaxis in children, resulting in reduced selection of *Nakaseomyces glabrata* [17]. Other studies in pediatric populations likewise demonstrate the predominance of non-*albicans* spp. [18,19]. This increased rate of *Candida* non-*albicans* spp. is of concern because this group frequently contains *Candida* species which are relatively or completely resistant to fluconazole [20].

The most common *Candida* spp. in our cohort was *C. albicans* (43.5%) followed by *C. parapsilosis* (16.6%) (Table 2), similar to other pediatric single- and multi-center studies [2,4,5,19,21,22], as well as to a local study in the Jerusalem general population [11]. Others, however, have demonstrated *C. parapsilosis* to be the leading pathogen in pediatric patients [23], especially neonates [23,24]. *C. albicans* is the most common pathogen due to its abundance on human skin and in the human gastrointestinal tract [25], whereas *C. parapsilosis* is associated with indwelling catheters and TPN, as it can form biofilm on intravascular device surfaces and on the hands of healthcare workers [26]. It is, therefore, possible that high rates of *C. parapsilosis* in pediatric patients are related to the high rate of central line usage. The proportion of isolates with high resistance rates to fluconazole, such as *Nakaseomyces glabrata* and *C. krusei*, was low in our cohort (8.4 and 4.6%, respectively), similar to a recent US pediatric study (7.7 and 5.3%, respectively), though higher than the rates observed in a European cohort over 11 years (3.5 and 2.2%, respectively) [22,23].

Of 132 *Candida* spp. in our study, 16 (12.1%) were fluconazole-resistant, which is at the higher end of rates reported in pediatric populations worldwide (2.9–12%) [2,4,19,23,24,27]. It is, however, important to note that the definition of resistance varies between studies; we defined fluconazole resistance as non-susceptibility (MICs above the susceptibility breakpoints). Other Israeli studies that include adult patients and use similar resistance definitions found fluconazole non-susceptibility rates to range between 6.8 and 12.1% [11,28,29]. Our relatively high overall fluconazole resistance rate may be related to patient backgrounds, with half immunocompromised and perhaps frequently exposed to fluconazole as prophylaxis or treatment.

Contrary to a European pediatric study, which observed increased fluconazole resistance [4], we found no such increase over the study years (15% in 2009–2017; 10.2% in 2018–2022), similar to other Australian and American pediatric studies [19,23].

Fluconazole resistance (MIC > 32 µg/mL) among *Nakaseomyces glabrata* species was 10% in our study. Such resistance is rarely reported in children: in multi-center and single-center studies in the US, resistance rates were 3.4 and 11.1%, respectively [2,23]. Other Israeli studies, which include adults and children, report a range of 4 to 11% [11,28,29].

It is important to note that the high rate of caspofungin resistance found among *Nakaseomyces glabrata* and *C. krusei* isolates (40 and 66.6%, respectively) is probably inaccurate due to the poor reliability of the caspofungin Etest, which was used in our study for these two species. Revised CLSI recommendations acknowledge the risk of Etest misclassifying caspofungin-susceptible *Nakaseomyces glabrata* and *C. krusei* as resistant, compared with broth microdilution (BMD), the reference method for determining antifungal susceptibility [30]. Another Israeli study of a large collection of *Nakaseomyces glabrata* isolates [31] found a low essential agreement rate (47.8%) between BMD and Etest in caspofungin testing. Caspofungin resistance among *Nakaseomyces glabrata* isolates, as determined by Etest, should thus be interpreted with caution. Validating resistance results with BMD may be advisable for invasive strains [31].

Unsurprisingly, the rate of *Candida* non-*albicans* was significantly higher in fluconazole-resistant episodes. Former pediatric cohorts have shown that previous exposure to azoles, especially fluconazole [27], is a major factor in fluconazole resistance. Others, such as older age [4], neutropenia [13,28], and chronic renal disease [13], were less consistently associated with resistance. The selective pressure exerted by widespread use of fluconazole may promote the proliferation of fluconazole-resistant *Candida* spp. [13], and previous exposure to fluconazole can promote development of resistance by generating overexpression or mutations of the fungal *ERG11* gene, which encodes lanosterol 14-alpha-demtheylase, the target enzyme for fluconazole [32,33]. The benefits of azole prophylaxis in high-risk patients [34] should, therefore, be balanced against risk of infection with fluconazole-resistant *Candida* isolates. Although we found no clear association between azole exposure or neutropenia and fluconazole resistance, the higher rate of exposure to fluconazole and prolonged severe neutropenia in patients who experienced fluconazole-resistant episodes support empirical treatment of suspected candidemia in these high-risk patients with echinocandins, in accordance with the 2016 IDSA invasive candidiasis guidelines [1].

The overall 30-day mortality in our study was 19.7%, higher than the usually reported range of 10.2 to 14.4% in children with candidemia, despite almost all the children receiving appropriate empirical therapy [2,4,19,35]. This finding is perhaps related to the relatively high proportion of immunocompromised patients with candidemia episodes (52%, Table 1), compared with 15.3–35.0% of such patients in other studies with lower mortality rates [2,23]. Specifically, in our study, there were higher proportions of patients with hematologic malignancy or HCT (43/127, 33.8%) compared with those in other investigations (14–16.8%) [2,23]. Moreover, the proportion of immunocompromised patients in our study increased after 2013 (from 41.1% in 2009–2013 to 59.2% in the latter period), paralleling the increase in mortality in those years (from 9.8 to 26.3%, respectively).

Thirty-day mortality risk factors in multivariate analysis were candidemia acquired in the PICU, presence of shock, and previous treatment with azoles or carbapenems—all factors indicating severe clinical condition. Risk factors for 30-day mortality in children found in other studies are hospitalization in PICU [22,27], exposure to systemic steroids, breakthrough candidemia [23], male gender, liver disease, and mucositis [19]. As delay in appropriate therapy can increase mortality, administration of empirical antifungals should be considered in patients with these risk factors in whom infection is suspected.

The limitations of our study are that it is an observational retrospective single-center study with a small sample size. The results are subject to bias and may not be generalizable to all pediatric patients with candidemia. The sample size is, however, comparable with other investigations [4,19,21,29], and a single-center study may better reflect local changes in epidemiology. In future, multi-center prospective studies shall analyze the epidemiology and risk factors for IC due to *Candida* spp. resistance to antifungal agents, and risk factors for mortality during the course of IC in pediatric population.

In conclusion, in this 14-year pediatric cohort study, *Candida* non-*albicans* spp. was the leading cause of candidemia. Rates of fluconazole resistance and 30-day mortality were in the higher range of those reported in children globally. Admission to PICU, septic shock, previous azole use, and wide-spectrum antibiotic exposure require consideration of empirical antifungal treatment when bloodstream infection is suspected, since they all increase candidemia mortality.

## Figures and Tables

**Table 1:** Factors associated with 30-day mortality in invasive candidiasis episodes.

	Total Episodes (*n*, %)	Episodes with 30 Days Survival (*n*, %)	Episodes with 30 Days Mortality (*n*, %)	*p* Value
Total patients (*n*)	127	102 (80.3)	25 (19.7)	
Female: Male	1.04	1.04	1.08	0.92
Age at first episode in years (median, IQR)	2.45 (0.36, 5.65)	2.5 (0.4, 5.3)	1.2 (0.05, 6.3)	0.28
Underlying diseases				
Immune-compromised patients	66 (51.9)	53 (52)	13 (52)	0.99
Allogeneic HCT	21 (16.5)	16 (15.7)	5 (20)	
Autologous HCT	6 (4.7)	5 (4.9)	1 (4)	
Hematological malignancy	16 (12.6)	15 (14.7)	1 (4)	
Solid tumors	14 (11)	13 (12.7)	1 (4)	
Others	9 (7.1)	4 (3.9)	5 (20)	
Prematurity	22 (17.3)	16 (15.7)	6 (24)	0.37
Other underlying disease	65 (51.2)	56 (54.9)	9 (36)	0.09
Abdominal surgery	18 (14.2)	11 (10.8)	7 (28)	0.049
Central venous catheter	104 (81.9)	82 (80.4)	22 (88)	0.56
Total parenteral nutrition	71 (55.9)	52 (51)	19 (76)	0.024
Number of hospitalization days in the last 3 months, median (IQR) (*n* = 124)	26.5 (10, 51)	27 (10, 50)	25 (8, 69.5)	0.92
Candidemia developed while in PICU	53 (41.7)	33 (32.4)	20 (80)	<0.001
Time from hospital admission to candidemia (first positive culture), days, median (IQR)	12 (5, 28)	11 (2, 26.5)	20 (8, 57.5)	0.017
Previous antifungal therapy	26 (20.5)	16 (15.7)	10 (40)	0.007
Azole	25 (19.7)	15 (14.7)	10 (40)	0.009
Amphotericin B	1 (0.8%)	1 (1)	0 (0)	1
Echinocandin	4 (3.1%)	3 (2.9)	1 (4)	1
Systemic antibiotics within a month preceding candidemia onset	114 (89.8)	90 (88.2)	24 (96)	0.46
Beta-lactam	105 (82.7)	83 (81.4)	22 (88)	0.53
Penicillins	59 (46.5)	46 (45.1)	13 (52)	0.53
Cephalosporins	45 (35.4)	40 (39.2)	5 (20)	0.072
Carbapenem	67 (52.8)	47 (46.1)	20 (80)	0.002
Aminoglycosides	38 (29.9)	28 (27.5)	10 (40)	0.22
Vancomycin	51 (40.2)	36 (35.3)	15 (60)	0.024
Colistin	7 (5.5)	5 (4.9)	2 (8)	0.6
Fluoroquinolones	21 (16.5)	17 (16.7)	4 (16)	1
Absolute neutrophil count 10^9^/L median (IQR)	4.5 (0.66, 10.5)	4.45 (0.5, 9.8)	6 (0.96, 15.7)	0.36
Severe neutropenia at the time of candidemia	30 (23.6)	25 (24.5)	5 (20)	0.63
Microbiological data				
*Candida* non-*albicans*	71 (55.9)	58 (56.8)	13 (52)	0.66
Poly-candidemia	4 (3.1)	3 (2.9)	1 (4)	1
Shock	27 (21.3)	13 (12.7)	14 (56)	0
Chronic disseminated candidiasis	15 (11.8)	14 (13.7)	1 (4)	0.3
CVC removal	46/104 (36.8)	39/82 (47.5)	7/22 (31.8)	0.15
Fluconazole resistance	10/121 (8.2)	8/98 (8.1)	2/23 (8.6)	1
Inappropriate empirical antifungal treatment	9 (7.1)	6 (5.9)	3 (12)	0.38
Duration of candidemia (days), median (IQR)	1 (0, 5)	0 (0, 4)	3 (0.5, 7.5)	0.05

HCT: hematopoietic stem cell transplantation, PICU: pediatric intensive care unit, CVC: central venous catheter.

**Table 2:** Resistance rates of Candida isolates to antifungals in invasive candidiasis episodes (presented as a number and rate of resistant isolates among total isolates tested for susceptibility).

*Candida* spp.	Fluconazole *n*/N (%)	Voriconazole *n*/N (%)	Caspofungin *n*/N (%)	Amphotericin B *n*/N (%)
*Candida albicans*	1/57 (1.7)	0/27 (0)	0/26 (0)	0/25 (0)
*Candida parapsilosis*	1/22 (4.5)	0/22	0/22 (0)	1/22 (4.5)
*Candida tropicalis*	1/17 (5.8)	1/17 (5.8)	0/17 (0)	0/17 (0)
*Nakaseomyces glabrata*	1/11 (10)	1/10 (10)	2/5 (40)	0/11 (0)
*Candida krusei*	6/6 (100)	0/6 (0)	4/6 (66.6)	0/6 (0)
Others ^a^	6/19 (31.5)	1/7 (14.2)	0/4 (0)	NA ^a^
Overall	16/132 (12.1)	3/89 (3.3)	6/80 (7.5)	1/80 (1.2)

NA; not applicable. ^a^ No breakpoints available.

**Table 3:** Multivariate analysis of risk factors for 30-day mortality in invasive candidiasis episodes.

Factors	Adjusted OR	95% CI	*p* Value
Candidemia developed while in PICU	7.07	1.89–26.39	0.004
Azole treatment in last month	4.43	0.98–19.98	0.05
Carbapenem in last month	4.77	1.27–17.79	0.02
Presence of shock	4.52	1.29–15.74	0.018

OR: odds ratio, CI: confidence interval, PICU: pediatric intensive care unit.

## Data Availability

Data are contained within the article.
